# Carbon Monoxide-Releasing Molecule-2 Ameliorates Particulate Matter-Induced Aorta Inflammation via Toll-Like Receptor/NADPH Oxidase/ROS/NF-*κ*B/IL-6 Inhibition

**DOI:** 10.1155/2021/2855042

**Published:** 2021-07-13

**Authors:** Thi Thuy Tien Vo, Chien-Yi Hsu, Yinshen Wee, Yuh-Lien Chen, Hsin-Chung Cheng, Ching-Zong Wu, Wei-Ning Lin, I-Ta Lee

**Affiliations:** ^1^School of Dentistry, College of Oral Medicine, Taipei Medical University, Taipei, Taiwan; ^2^Division of Cardiology, Department of Internal Medicine, School of Medicine, College of Medicine, Taipei Heart Institute, Taipei Medical University, Taipei, Taiwan; ^3^Division of Cardiology and Cardiovascular Research Center, Department of Internal Medicine, Taipei Medical University Hospital, Taipei, Taiwan; ^4^Department of Pathology, University of Utah, Salt Lake City, UT, USA; ^5^Department of Anatomy and Cell Biology, College of Medicine, National Taiwan University, Taipei, Taiwan; ^6^Department of Dentistry, Taipei Medical University Hospital, Taipei, Taiwan; ^7^Graduate Institute of Biomedical and Pharmaceutical Science, Fu Jen Catholic University, New Taipei City, Taiwan

## Abstract

Particulate matter (PM), a major air pollutant, may be associated with adverse cardiovascular effects. Reactive oxygen species- (ROS-) dependent proinflammatory cytokine production, such as interleukin-6 (IL-6), is a possible underlying mechanism. Carbon monoxide- (CO-) releasing molecule-2 (CORM-2) which liberates exogenous CO can exert many beneficial effects, particularly anti-inflammation and antioxidant effects. The purpose of this study was to explore the protective effects and underpinning mechanisms of CORM-2 on PM-induced aorta inflammation. Here, human aortic vascular smooth muscle cells (HASMCs) were utilized as *in vitro* models for the assessment of signaling pathways behind CORM-2 activities against PM-induced inflammatory responses, including Toll-like receptors (TLRs), NADPH oxidase, ROS, nuclear factor-kappa B (NF-*κ*B), and IL-6. The modulation of monocyte adherence and HASMC migration, that are two critical cellular events of inflammatory process, along with their regulators, including intercellular adhesion molecule-1 (ICAM-1), vascular cell adhesion molecule-1 (VCAM-1), and matrix metalloproteinase-2 (MMP-2) and MMP-9, in response to PM by CORM-2, were further evaluated. Finally, mice experiments under different conditions were conducted for the *in vivo* evaluation of CORM-2 benefits on the expression of inflammatory molecules including IL-6, ICAM-1, VCAM-1, MMP-2, and MMP-9. Our results found that PM could induce aorta inflammation *in vitro* and *in vivo*, as evidenced by the increase of IL-6 expression that was regulated by the TLR2 and TLR4/NADPH oxidase/ROS/NF-*κ*B signaling pathway, thereby promoting ICAM-1- and VCAM-1-dependent monocyte adhesion and MMP-2- and MMP-9-dependent HASMC migration. Importantly, our experimental models demonstrated that CORM-2-liberated CO effectively inhibited the whole identified PM-induced inflammatory cascade in HASMCs and tissues. In conclusion, CORM-2 treatment may elicit multiple beneficial effects on inflammatory responses of aorta due to PM exposure, thereby providing therapeutic value in the context of inflammatory diseases of the cardiovascular system.

## 1. Introduction

Nowadays, air pollution is a global challenge with particulate matter (PM) being one of its dominant components. PM can be described as a complex mixture of extremely small particles and liquid droplets that get into the air and cause various health effects upon inhalation [1]. Many lines of evidence have reported the association between PM exposure with adverse cardiovascular effects [2, 3]. A growing body of studies have shed light on the mechanisms behind PM-induced cardiovascular diseases (CVDs), among which reactive oxygen species- (ROS-) dependent proinflammatory cytokine production has been widely accepted [3]. Subclinically and clinically, vascular inflammation is a milestone in the development of many CVDs [4]. Despite the importance of vascular smooth muscle cells (VSMCs) in the progression of vascular inflammation [5], their regulation remains understudied as compared to that of endothelial cells or inflammatory cells, demanding much more research.

Interleukin-6 (IL-6), a pleiotropic cytokine, has been implicated in the pathogenesis of various CVDs [6]. As major component of vasculature, VSMCs have been shown to produce IL-6 in response to different stimuli, particularly angiotensin II, which is a well-known cardiovascular risk factor [7, 8]. A plausible mechanism linking IL-6 with CVDs is oxidative stress, which may result from the overproduction of ROS such as vascular superoxide [9]. Nicotinamide adenine dinucleotide phosphate (NADPH) oxidase is known as the “factory” for superoxide generation [10]. In fact, angiotensin II-induced IL-6 increase in VSMCs was markedly prevented by the addition of superoxide pharmacologic scavenger [11], NADPH oxidase inhibitor [11], or NADPH oxidase subunit antibody [12], suggesting that ROS elevation, particularly NADPH oxidase-derived superoxide, may regulate IL-6 production. Moreover, the nuclear factor-kappa B (NF-*κ*B) transcription factor, known as a major inducer of IL-6 transcription and release [13], was found as a mediator for vascular superoxide-dependent IL-6 expression in human aortic smooth muscle cells (HASMCs) [14]. Besides, the activation of Toll-like receptors (TLRs), a family of pattern recognition molecules, may also trigger different signaling pathways, including NF-*κ*B [15]. To date, TLR2 and TLR4 have been increasingly recognized to share common pathways, eliciting synergetic effects on inflammatory responses such as vascular inflammation [5]. Previous literature reported that IL-6 was modulated by TLR2-dependent NF-*κ*B activation in VSMCs [16], and so was TLR4 [17]. Collectively, IL-6-driven vascular inflammation is tightly regulated by a complicated network of signaling pathways. While the capacity of PM to induce oxidative stress and IL-6 production has been revealed in earlier studies [2, 3], the underlying mechanisms in vasculature remain unclear. In this study, PM was hypothesized to cause IL-6-driven vascular inflammation through the coordination of TLR2 and TLR4, NADPH oxidase-derived ROS, and NF-*κ*B signaling molecules.

Although carbon monoxide (CO) has been long recognized as a “silent killer” due to its greater affinity for hemoglobin than oxygen, the intriguing role of CO as an endogenous signaling molecule has been appreciated in recent years. The biological activities of CO have been widely investigated, and they have been reported to exert multiple beneficial effects with potential therapeutic applications, particularly anti-inflammation and antioxidant [18]. A group of transition metal carbonyl complexes, known as carbon monoxide-releasing molecules (CORMs) which can release CO upon transformation, has been developed as a superior alternative to CO administration [19, 20]. The lipid-soluble metal carbonyl complex tricarbonyldichlororuthenium (II) dimer, also termed CORM-2, serves as a promising candidate in CO-based therapy [21, 22]. Our previous studies found that the liberation of CO from CORM-2 was able to reduce angiotensin II-induced inflammatory responses in HASMCs by inhibiting the IL-6 signaling pathway [14], or it could elicit protective effects against PM-induced lung inflammation in a TLR2- and TLR4-mediated ROS-dependent manner [23]. Herein, CO released from CORM-2 was assumed to exhibit anti-inflammatory effects on vascular inflammation in response to PM via the inhibition of the IL-6 axis.

One of the hallmarks during vascular inflammation is the adhesion of leukocytes to vascular endothelium that is a multistep cascade regulated by different families of surface-expressed cell adhesion molecules such as intercellular adhesion molecule-1 (ICAM-1) and vascular cell adhesion molecule-1 (VCAM-1) [24]. In addition to endothelial cells, several lines of evidence have also reported the expression of ICAM-1 and VCAM-1 on VSMCs, which may correlate with mononuclear cell infiltration and contribute to the progression of vascular inflammation [25]. Previous findings have suggested the potential effects of CORM-2 in the alleviation of inflammation in various experimental models through the inhibition of these adhesion molecules [26–28]; however, there is a lack of empirical evidences regarding VSMC models, especially under PM exposure. As a result, this work proposed that CORM-2 could exert beneficial effects on PM-induced vascular inflammation by inhibiting monocyte adhesion regulated by ICAM-1 and VCAM-1.

Matrix metalloproteinases (MMPs) are a family of endopeptidases capable of degrading components of the extracellular matrix (ECM). Uncontrolled MMP activity can result in the breakdown of ECM, enabling cell migration [29]. Of various MMP members, gelatinases consisting of gelatinase A (MMP-2) and gelatinase B (MMP-9) play important roles in the migration of various types of vascular cells, including VSMCs, thereby contributing to the pathogenesis of vascular diseases [30]. Earlier investigation estimated a strong association between the concentration of PM with a diameter ≤ 2.5 *μ*m (PM2.5) with the increase of MMP-2 and MMP-9 as circulating biomarkers relevant to atherosclerotic plaque vulnerability, thrombogenicity, and inflammation in healthy adults [31]. As CORM-2 was previously demonstrated to mitigate angiotensin II-induced HASMC migration in an MMP-9-dependent manner [14], the present study further presumed that MMP-2- and MMP-9-dependent vascular cell migration may be an additional target of CORM-2 for the impediment of PM-induced vascular inflammation.

In this study, HASMCs were utilized as *in vitro* models for the identification of signaling pathways behind CORM-2 effects against PM-induced vascular inflammatory responses, and mice experiments were implemented for the *in vivo* assessment of CORM-2 protective benefits on PM-induced aorta inflammation. The purpose of our work was to explore the potentials of CORM-2 in the inhibition of PM-induced IL-6 expression, monocyte adhesion, and HASMC migration, which are hallmarks of vascular inflammation (e.g., aorta inflammation) implicated in many VCDs, as well as the underlying mechanisms.

## 2. Materials and Methods

### 2.1. Reagents

Urban PM (SRM 1648a) and tricarbonyldichlororuthenium (II) dimer (CORM-2) were purchased from Sigma-Aldrich (St. Louis, MO, USA). The inactive variant iCORM-2 was generated through the inactivation of CORM-2 by replacing carbonyl groups with DMSO, as described in a previous study [32]. Apocynin (APO), N-acetyl-L-cysteine (NAC), diphenyleneiodonium chloride (DPI), and helenalin (HLN) were obtained from Sigma-Aldrich (St. Louis, MO, USA); whereas BCECF-AM (2′,7′-bis-(2-carboxyethyl)-5-(and-6)-carboxyfluorescein, acetoxymethyl ester) was taken from Invitrogen (CA, USA).

### 2.2. Cell Culture

HASMCs, purchased as cryopreserved tertiary cultures from Cascade Biologics, Inc. (OR), were grown in culture flasks in smooth muscle cell growth medium M231 (Cascade Biologics, Inc.) supplemented with fetal bovine serum (FBS, 5%), human epidermal growth factor (10 ng/ml), human basic fibroblast growth factor (3 ng/ml), insulin (10 mg/ml), penicillin (100 U/ml), streptomycin (100 pg/ml), and Fungizone (1.25 mg/ml) at 37°C in a humidified 5% CO_2_ atmosphere. The growth medium was changed every other day until confluence; then, cells were passaged by division between four Petri dishes and grown to confluence again. Cells between passages 3 and 7 were utilized for incubation with PM and/or CORM-2 under different conditions. The purity of the HASMC culture was verified by immunostaining with a monoclonal antibody against smooth muscle-specific *α*-actin.

### 2.3. Cell Viability

Following 24 h incubation with different concentrations of PM (10, 25, 50, 100, and 200 *μ*g/cm^2^) or CORM-2 (10, 25, 50, and 100 *μ*M), the cell viability of HASMCs in response to each exposure was determined by using the PrestoBlue Cell Viability Reagent (Invitrogen, CA, USA) in accordance to the manufacturer's protocol.

### 2.4. Transient Transfection with Human Small Interfering RNAs (siRNAs)

In our laboratory, the transient transfection of siRNAs was generally carried out using the Lipofectamine 2000 Transfection Reagent (Invitrogen, CA, USA) [33, 34]. In this study, human scrambled, TLR2, TLR4, p47^phox^, p67^phox^, p65, p50, and IL-6 siRNAs from Santa Cruz Biotechnology, Inc. (Santa Cruz, CA, USA) were utilized.

### 2.5. Western Blot

After incubation under various experimental conditions, Western blot was implemented to identify the protein expression for the molecules of interest, as described in our previous publications [33, 35]. Briefly, HASMCs were successively washed, scraped, collected, and lysed to obtain the lysates. The lysates were then centrifuged to yield the whole cell extract for further steps, sequentially including sample preparation, loading and running SDS-PAGE gel electrophoresis with 10% running gel, transferring the proteins from the gels to the nitrocellulose membranes, and immunodetection of proteins. For the final step, anti-*β*-actin, anti-IL-6, anti-TLR2, anti-TLR4, anti-GAPDH, anti-G*α*s, anti-p47^phox^, anti-phospho-p65, anti-VCAM-1, anti-ICAM-1, anti-MMP-2, and anti-MMP-9 antibodies were purchased from Santa Cruz Biotechnology, Inc. (Santa Cruz, CA). The membranes were incubated overnight at 4°C with these antibodies at a dilution of 1 : 1000 in TTBS, and the immunoreactive bands were then visualized by using secondary antibodies and enhanced chemiluminescence reagents.

### 2.6. Real-Time PCR

After incubation under various experimental conditions, real-time PCR was performed to determine the mRNA levels for the molecules of interest, as presented in our previous studies [33–35]. Briefly, total RNAs were extracted and reversely transcribed into cDNAs for analysis using SYBR Green PCR reagents (Applied Biosystems, Branchburg, NJ, USA). In this study, primers specific for IL-6, TLR2, TLR4, ICAM-1, VCAM-1, MMP-2, MMP-9, TIMP-1, TIMP-2, and GAPDH mRNAs were utilized.

### 2.7. Measurement of IL-6 Generation

After incubation under various experimental conditions, the media of HASMC culture were collected, and the IL-6 secretion was assayed using an IL-6 ELISA kit (ab46027) (Abcam, Cambridge, UK).

### 2.8. Measurement of Intracellular ROS Generation and NADPH Oxidase Activity

The protocol utilized in our laboratory to determine the intracellular ROS generation and NADPH oxidase activity was clearly reported in previous work [33, 35]. Briefly, the levels of intracellular ROS in HASMCs under various experimental conditions were measured using the CellROX® Green Reagent, and the fluorescence intensity was analyzed by a FACScan flow cytometer (BD Biosciences, San Jose, CA) at 485 nm excitation and 520 nm emission. Meanwhile, the activity of NADPH oxidase was monitored based on the measurement of chemiluminescence using the Appliskan luminometer (Thermo Fisher Scientific).

### 2.9. Cell Fraction Isolation

For the isolation of proteins from three cellular fractions (the nucleus, the cytosol, and the membrane), the Nuclear/Cytosol Fractionation Kit (Cat. #K266) and the Membrane Protein Extraction Kit (Cat. #K268) from BioVision (Milpitas, CA, USA) were utilized in our laboratory [33, 35].

### 2.10. Analysis of Luciferase Reporter Gene Activity

An NF-*κ*B reporter kit (BPS Bioscience, San Diego, CA, USA) was utilized to measure the transcriptional activity of NF-*κ*B in HASMCs under various experimental conditions, as described in our previous work [35].

### 2.11. Migration Assay

HASMCs were cultured to confluence in 10 cm dishes and starved with serum-free M231 for 24 h. The monolayer cells were scratched manually with a blade to create extended and definite scratches in the center of the dishes with a bright and clear field. The detached cells were removed by washing once with PBS. Serum-free M231 with or without PM was added to each dish as indicated after pretreatment with the inhibitors for 2 h, containing a DNA synthesis inhibitor hydroxyurea (10 *μ*M) during the period of incubation. Images of migratory cells from the scratched boundary were observed and acquired at 0 and 24 h with a digital camera and a light microscope (Olympus, Japan). The number of migratory cells was counted from the resulting four phase images for each point and then averaged for each experimental condition. The data were generated from three separate assays.

### 2.12. Adhesion Assay

THP-1 cells were labeled with 10 *μ*M BCECF-AM at 37°C for 1 h in RPMI-1640 medium (Gibco BRL, Grand Island, NY) and subsequently washed by centrifugation. Confluent HASMCs were incubated with THP-1 cells (2 × 10^6^ cells/ml) at 37°C for 1 h. Nonadherent THP-1 cells were removed and plates were gently washed with PBS. The numbers of adherent THP-1 cells were counted under four fields per 200x high-power field well using a fluorescent microscope (Zeiss, Axiovert 200M). Four randomly chosen high-power fields were counted per well.

### 2.13. Animal Care and Experimental Procedure

Male BALB/c mice aged 6-8 weeks were purchased from the National Laboratory Animal Center (Taipei, Taiwan) and were handled according to the Guidelines of the Animal Care Committee of Chang Gung University and NIH Guides for the Care and Use of Laboratory Animals. Mice were anesthetized with ethyl ether and placed individually on a board in a near vertical position, and the tongues were withdrawn by using lined forceps. Twenty *μ*l of PM suspension (8 mg/ml) was placed posteriorly in the throat and aspirated into the lungs. Control mice were administrated sterile 0.1% bovine serum albumin. Mice regained consciousness after 15 min. Mice were given i.v. one dose of NAC (2 mg/kg), DPI (2 mg/kg), HLN (2 mg/kg), or CORM-2 (8 mg/kg) prior to PM treatment and were sacrificed after 72 h. Thoracic aorta tissues were prepared for analysis by Western blot and real-time PCR to determine the protein expression and mRNA levels of IL-6, MMP-2, MMP-9, VCAM-1, and ICAM-1.

### 2.14. Statistical Analysis

The quantitative data were presented as mean ± S.E.M. Analysis of data was performed with one-way ANOVA followed by Tukey's post hoc test using the GraphPad Prism program (GraphPad, San Diego, CA, USA). Statistical significance was set at *p* value < 0.05.

## 3. Results

### 3.1. PM Induces IL-6 Secretion in HASMCs

**Figure 1:**
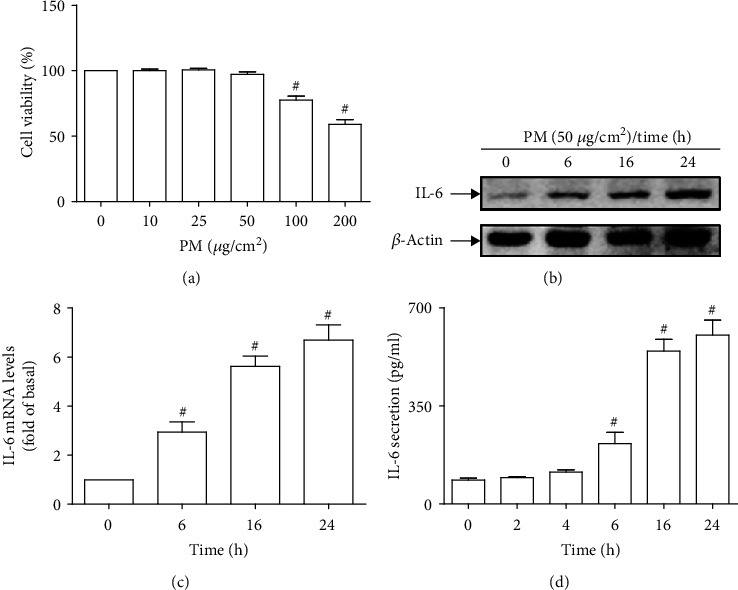
PM induces IL-6 expression and secretion in HASMCs. HASMCs were treated with various concentrations of PM for 24 h, and then the cell viability was measured. HASMCs were treated with PM of 50 *μ*g/cm^2^ for designated times, and then the IL-6 (a) protein expression, (b) mRNA levels, and (c) secretion were determined. Data are presented as mean ± S.E.M. of four independent experiments. ^#^*P* < 0.01, as compared with control.

As shown in Figure 1(a), PM yielded no adverse effects on the cell viability of HASMCs at concentrations not exceeding 50 *μ*g/cm^2^; therefore, PM at a concentration of 50 *μ*g/cm^2^ were utilized for subsequent experiments. The expression and release of IL-6 has been reported to link to local vascular inflammation, including inflammation of the aorta [36]. In this study, PM could lead to IL-6 protein expression, mRNA levels, and secretion in HASMCs in a time-dependent manner (Figures 1(b) – 1(d)), suggesting the capacity of PM to induce aortic inflammatory responses via IL-6 signaling.

### 3.2. PM Induces IL-6 Expression via TLR2 and TLR4

**Figure 2:**
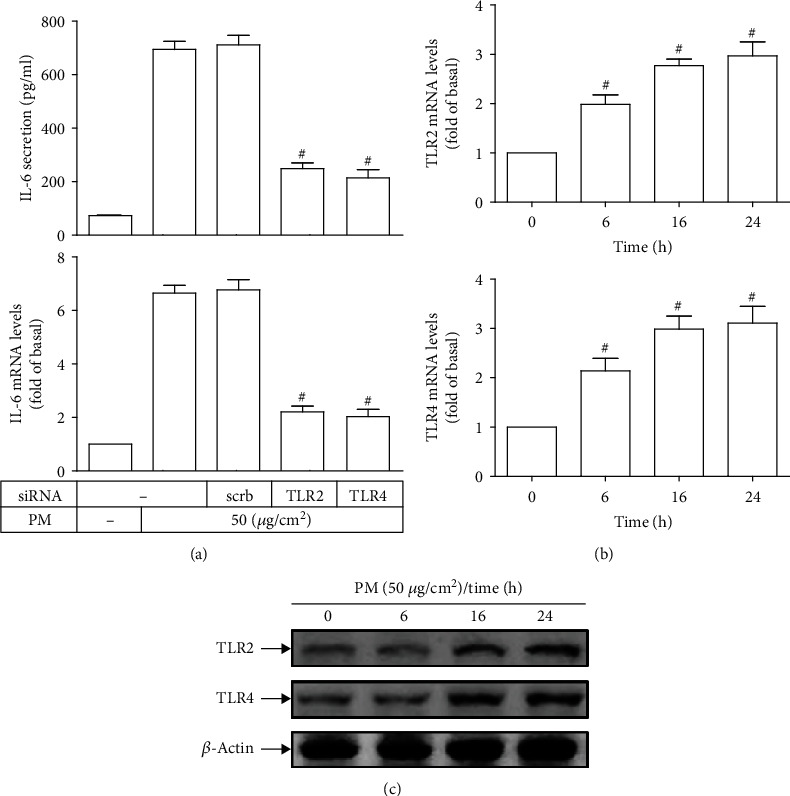
PM induces IL-6 expression via TLR2 and TLR4 in HASMCs. (a) HASMCs were transfected with siRNA of scrambled (scrb), TLR2, or TLR4, followed by PM incubation for 24 h. The IL-6 secretion and mRNA levels were measured. Cells were treated with PM for designated times, and then the (b) mRNA levels and (c) protein expression of TLR2 and TLR4 were determined. Data are presented as mean ± S.E.M. of four independent experiments. ^#^*P* < 0.01, as compared with the cells exposed to PM plus scrb siRNA (a). ^#^*P* < 0.01, as compared with control (b).

IL-6 can issue a warning signal in response to both infection and noninfectious tissue damage which may be recognized by TLRs such as TLR2 and TLR4 [5, 37]. As shown in Figure 2(a), IL-6 mRNA levels and secretion following PM incubation were significantly reduced in HASMCs transfected with siRNAs of TLR2 or TLR4 as compared with those exposed to PM alone. In addition, the mRNA levels and protein expression of TLR2 and TLR4 were markedly induced by PM in a time-dependent manner (Figures 2(b) and 2(c)). Altogether, our results suggested that PM exposure could trigger the expression of TLR2 and TLR4, resulting in the release of IL-6 in HASMCs.

### 3.3. PM Induces IL-6 Expression via NADPH Oxidase/ROS

**Figure 3:**
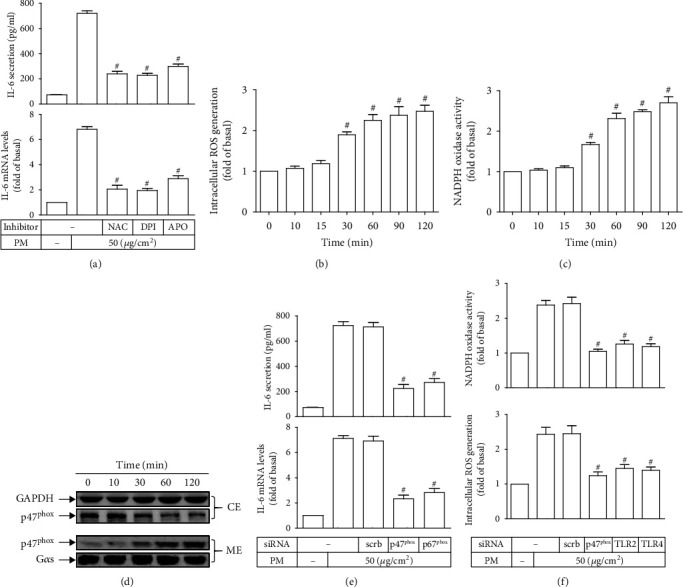
PM induces IL-6 expression via NADPH oxidase-derived ROS generation. (a) HASMCs were pretreated with either NAC (10 mM), DPI (1 *μ*M), or APO (100 *μ*M) for 2 h, followed by PM incubation for 24 h. The IL-6 secretion and mRNA levels were measured. Cells were treated with PM for designated times, and then the (b) ROS generation and (c) NADPH oxidase activity were determined. (d) Cells were incubated with PM for different intervals. The cytosolic and membrane fractions were prepared, and then the protein expression of p47^phox^ in each fraction was analyzed by Western blot (note: CE = cytosolic extraction; ME = membrane extraction). (e) Cells were transfected with siRNA of scrambled (scrb), p47^phox^, or p67^phox^, followed by PM incubation for 24 h. The IL-6 secretion and mRNA levels were measured. (f) Cells were transfected with siRNA of scrambled (scrb), p47^phox^, TLR2, or TLR4, and subsequently incubated with PM for 2 h. The ROS generation and NADPH oxidase activity were determined. Data are presented as mean ± S.E.M. of three independent experiments. ^#^*P* < 0.01, as compared with the cells exposed to PM alone (a) or PM plus scrb siRNA (e, f). ^#^*P* < 0.01, as compared with control (b).

Increase in ROS generation, particularly through the activation of NADPH oxidase, might serve as upstream of IL-6 expression in different vascular cell types [9]. Indeed, our data indicated that pretreatment of HASMCs with either NAC (10 mM), DPI (1 *μ*M), or APO (100 *μ*M), could significantly inhibit PM-induced IL-6 secretion and mRNA levels (Figure 3(a)). NAC is a synthetic scavenger of ROS (i.e., superoxide), whereas DPI and APO are pharmacologic inhibitors of NADPH oxidase. The concentrations of inhibitors applied in our study were based on concentrations of effective inhibition reported in previous studies [38, 39]. In addition, our study found that intracellular ROS generation in HASMCs was markedly elevated as early as 30 min upon PM incubation, and continuously increased over time (Figure 3(b)). Similar results were also obtained for NADPH oxidase activity (Figure 3(c)), suggesting that NADPH oxidase was a major source of PM-induced intracellular ROS production in HASMCs. The translocation from the cytosol to the membrane of p47^phox^, known as a pivotal cytosolic component of NADPH oxidase [10], were time-dependently detected in PM-exposed HASMCs by Western blot (Figure 3(d)), further demonstrating the activation of NADPH oxidase in these vascular cells upon PM stimulation. In fact, the intracellular ROS generation and NADPH oxidase activity caused by PM were significantly alleviated in HASMCs transfected with p47^phox^ siRNA as compared to those transfected with scrambled (control) siRNA (Figure 3(f)). Notably, as shown in Figure 3(e), the transfection of HASMCs with p47^phox^ siRNA could effectively attenuate PM-induced IL-6 secretion and mRNA levels, as comparable as cells transfected with siRNA of p67^phox^, another important cytosolic protein of NADPH oxidase [10]. Collectively, these data suggested that PM was able to trigger NADPH oxidase activation in HASMCs, resulting in ROS overproduction, thereby leading to IL-6 release. Since NADPH oxidase, ROS, TLR2, and TLR4 could be involved in PM-induced IL-6 expression, their relationship was further investigated. As shown in Figure 3(f), PM-induced intracellular ROS generation and NADPH oxidase activity were evidently reduced in HASMCs transfected with TLR2 or TLR4 siRNAs, indicating that the activation of TLR2 and TLR4 may serve as upstream of the increase in NADPH oxidase-derived ROS in these vascular cells under PM insult.

### 3.4. PM Induces IL-6 Expression through NADPH Oxidase/ROS/NF-*κ*B

**Figure 4:**
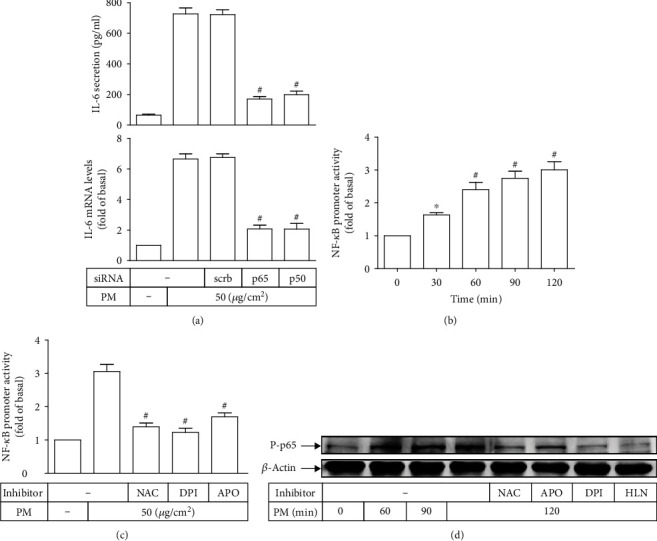
PM induces IL-6 expression through ROS-dependent NF-*κ*B activation. (a) HASMCs were transfected with siRNA of scrambled (scrb), p65, or p50, followed by PM incubation for 24 h. The IL-6 secretion and mRNA levels were measured. (b) Cells were treated with PM for designated times, and then the NF-*κ*B promoter activity was measured. (c) Cells were pretreated with NAC (10 mM), DPI (1 *μ*M), or APO (100 *μ*M) for 2 h, followed by PM incubation for 2 h. The NF-*κ*B promoter activity was measured. (d) Cells were pretreated with NAC (10 mM), APO (100 *μ*M), DPI (1 *μ*M), or HLN (1 *μ*M) for 2 h, and subsequently incubated with PM for different intervals. The phospho-p65 protein expression was determined. Data are presented as mean ± S.E.M. of three independent experiments. ^#^*P* < 0.01, as compared with the cells exposed to PM plus scrb siRNA (a) or PM alone (c). ^∗^*P* < 0.05 and ^#^*P* < 0.01, as compared with control (b).

The NF-*κ*B transcription factor may act as an important signal integrator involved in the process of vascular inflammation, and IL-6 is one of the most highly induced NF-*κ*B-dependent cytokines in various vascular cell types [13]. In line with literature, our results found that IL-6 secretion and mRNA levels following PM incubation were significantly reduced in HASMCs transfected with siRNAs of NF-*κ*B subunits, p65 or p50, as compared with those transfected with scrambled siRNA (Figure 4(a)), suggesting the role of NF-*κ*B for the regulation of PM-induced IL-6 expression in these vascular cells. Besides, there was a time-dependent increase of NF-*κ*B promoter activity in PM-treated HASMCs (Figure 4(b)), indicating that PM was able to upregulate NF-*κ*B transcriptional activity in these cells. To explore the signaling pathway mediating NF-*κ*B, HASMCs were treated with ROS scavenger (NAC) or NADPH oxidase inhibitors (DPI or APO) prior to PM incubation, then followed by the assessment of NF-*κ*B promoter activity. As shown in Figure 4(c), pretreatment with either NAC, DPI, or APO effectively inhibited PM-induced NF-*κ*B transcriptional upregulation in HASMCs. This finding was further confirmed by Western blot analysis in which PM could induce p65 phosphorylation in HASMCs in a time-dependent manner (Figure 4(d)). Such phosphorylation was also evidently attenuated by pretreatment with either NAC, DPI, or APO, as comparable as pretreatment with the selective inhibitor of NF-*κ*B, namely, HLN (Figure 4(d)). These results demonstrated that PM was able to lead to NADPH oxidase-derived ROS-dependent NF-*κ*B activation. Taken together, our data proposed that the NADPH oxidase/ROS/NF-*κ*B signaling pathway may regulate PM-induced IL-6 expression in HASMCs.

### 3.5. CORM-2 Reduces PM-Induced IL-6 Expression

**Figure 5:**
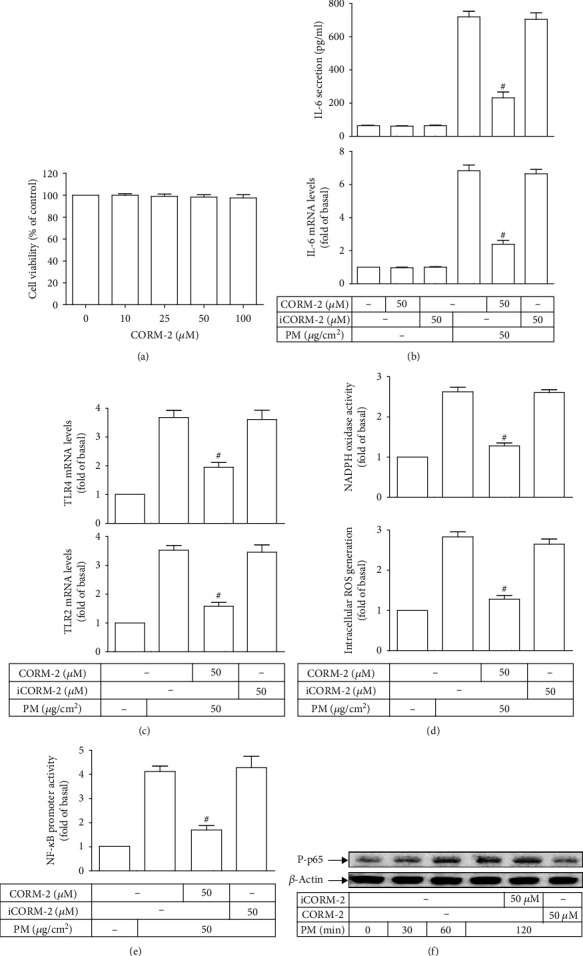
CORM-2 reduces PM-induced IL-6 expression in HASMCs. (a) HASMCs were treated with various concentrations of CORM-2 for 24 h, and then the cell viability was measured. (b) Cells were pretreated with iCORM-2 or CORM-2 of 50 *μ*M, followed by PM incubation for 24 h. (c) The IL-6 secretion and mRNA levels were measured. The TLR2 and TLR4 mRNA levels were measured. Cells were pretreated with iCORM-2 or CORM-2 and subsequently incubated with PM for 2 h. (d) The ROS generation and NADPH oxidase activity, as well as (e) the NF-*κ*B promoter activity were determined. (f) Cells were pretreated with iCORM-2 or CORM-2, and then incubated with PM for different intervals. The phospho-p65 protein expression was examined. Data are presented as mean ± S.E.M. of three independent experiments. ^#^*P* < 0.01, as compared with the cells exposed to PM alone.

As shown in Figure 5(a), CORM-2 yielded no deleterious impact on the cell viability of HASMCs at all examined concentrations, suggesting its nontoxicity on these cells. The concentration of CORM-2 at 50 *μ*M was utilized for subsequent experiments. To determine the effects of CORM-2 on PM-induced IL-6 expression, HASMCs were treated with either CORM-2 or its inactive variant (iCORM-2) prior to PM incubation for IL-6 measurement. Our data found that both IL-6 secretion and mRNA levels were markedly reduced in cells pretreated with CORM-2 as compared with those exposed to PM only; however, there was no alteration in cells treated with iCORM-2 (Figure 5(b)). This finding postulated that exogenous CO released by CORM-2 rather than CORM-2 itself may exert inhibitory effects on PM-induced IL-6 expression in HASMCs. To further unveil the signaling pathway underpinning CORM-2 potency, TLR2 and TLR4 mRNA levels, intracellular ROS generation, NADPH oxidase activity, NF-*κ*B promoter activity, and p65 phosphorylation were evaluated. Interestingly, our results showed that all measurements of those signaling molecules following PM incubation were significantly alleviated in HASMCs pretreated with CORM-2, but not iCOMR-2, as compared to cells exposed to PM alone (Figures 5(c) – 5(f)). Thus, our findings collectively demonstrated that CORM-2-liberated CO could attenuate PM-induced IL-6 expression in HASMCs through the inhibition of TLR2, TLR4, NADPH oxidase, ROS, and NF-*κ*B network.

### 3.6. CORM-2 Inhibits PM-Induced ICAM-1 and VCAM-1 Expression and Monocyte Adhesion

**Figure 6:**
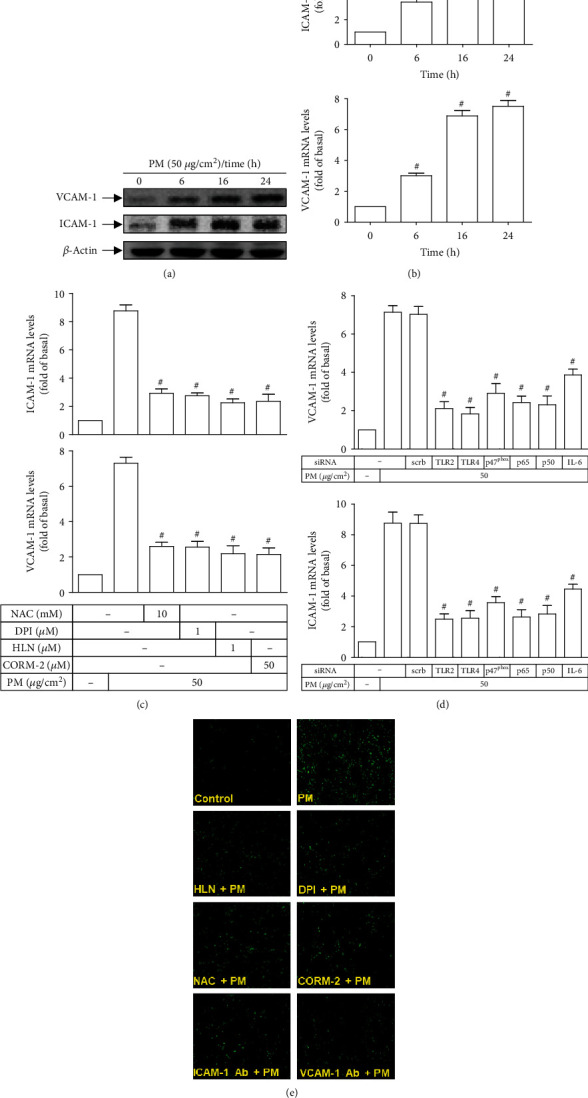
CORM-2 inhibits PM-induced ICAM-1 and VCAM-1 expression and monocyte adhesion in HASMCs. HASMCs were treated with PM for designated times, and then the VCAM-1 and ICAM-1 (a) protein expression and (b) mRNA levels were determined. (c) Cells were pretreated with NAC (10 mM), DPI (1 *μ*M), HLN (1 *μ*M), or CORM-2 (50 *μ*M) for 2 h, followed by PM incubation for 24 h. The VCAM-1 and ICAM-1 mRNA levels were measured. (d) Cells were transfected with siRNA of scrambled (scrb), TLR2, TLR4, p47^phox^, p65, p50, or IL-6, and subsequently incubated with PM for 24 h. The VCAM-1 and ICAM-1 mRNA levels were analyzed. (e) Cells were pretreated with HLN (1 *μ*M), DPI (1 *μ*M), NAC (10 mM), CORM-2 (50 *μ*M), ICAM-1 neutralizing antibody (10 *μ*g/ml), or VCAM-1 neutralizing antibody (10 *μ*g/ml) for 2 h prior to incubation with PM for 24 h. The adherence of THP-1 monocytic cells was measured. All figures are representative of three independent experiments performed in duplicate. Data are presented as mean ± S.E.M. of three independent experiments. ^#^*P* < 0.01, as compared with control (b). ^#^*P* < 0.01, as compared with the cells exposed to PM alone (c) or PM plus scrb siRNA (d).

Since leukocyte adhesion is one of critical steps in the process of vascular inflammation [24], the effects of CORM-2 on leukocyte adhesion was further investigated in order to clarify the protective benefits of CORM-2 on HASMCs under PM stimulation. Firstly, the protein expression and mRNA levels of two important cell adhesion molecules for leukocyte adherence, that is ICAM-1 and VCAM-1 [24], in HASMCs upon PM incubation for indicated times were measured. The results showed that both ICAM-1 and VCAM-1 protein expression and mRNA levels were significantly enhanced as early as 6 h following PM exposure, which were continuously increased over time (Figures 6(a) and 6(b)). Next, the possible mechanisms underlying PM-induced ICAM-1 and VCAM-1 increase as well as the effects of CORM-2 on the expression of these molecules were examined. As shown in Figure 6(c), pretreatment of HASMCs with either inhibitor of ROS (NAC), NADPH oxidase (DPI), or NF-*κ*B (HLN) effectively reduced PM-induced ICAM-1 and VCAM-1 mRNA levels, suggesting that the PM-induced ICAM-1 and VCAM-1 expression could be regulated by the NADPH oxidase/ROS/NF-*κ*B axis. This assumption was further confirmed by the data regarding PM-induced ICAM-1 and VCAM-1 mRNA levels of HASMCs transfected with siRNAs of TLR2, TLR4, p47^phox^, p65, p50, or IL-6.  Figure 6(d) shows that all of the transfections were able to significantly attenuate PM-induced ICAM-1 and VCAM-1 mRNA levels in HASMCs, implying that ICAM-1 and VCAM-1 may be the downstream targets of the TLR2 and TLR4/NADPH oxidase/ROS/NF-*κ*B/IL-6 signaling pathway. Importantly, HASMCS pretreated with CORM-2 exhibited pronounced reduction in PM-induced ICAM-1 and VCAM-1 mRNAs as compared with those exposed to PM alone (Figure 6(c)). Finally, the adherence capacity of human monocytic cells THP-1 on HASMCs under different experimental conditions were evaluated. As shown in Figure 6(e), PM could strongly induce the adherence of THP-1 cells on HASMCs, which was evidently mitigated by pretreatment with either NAC, DPI, HLN, or ICAM-1- or VCAM-1-neutralizing antibody, suggesting the regulatory role of NADPH oxidase/ROS/NF-*κ*B pathway in ICAM-1- and VCAM-1-dependent monocyte adhesion. Notably, HASMCs pretreated with CORM-2 prior to PM incubation also exhibited markedly lesser THP-1 cell adherence than those exposed to PM alone (Figure 6(e)). Collectively, CORM-2 could inhibit PM-induced ICAM-1 and VCAM-1 expression and subsequent monocyte adhesion in HASMCs.

### 3.7. PM Induces MMP-2 and MMP-9 Expression and Cell Migration via TLR2, TLR4, NADPH Oxidase, NF-*κ*B, and IL-6 in HASMCs

**Figure 7:**
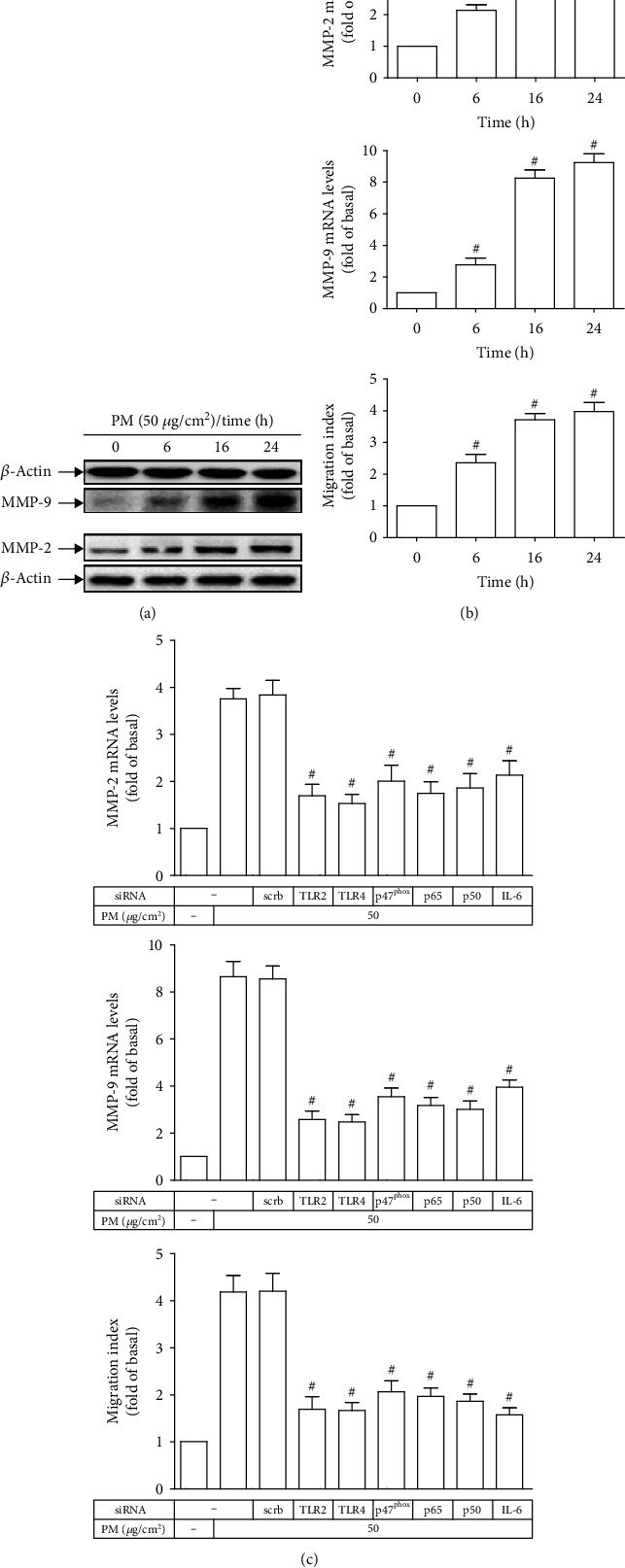
PM induces MMP-2 and MMP-9 expression and cell migration via TLR2, TLR4, NADPH oxidase, NF-*κ*B, and IL-6 in HASMCs. (a, b) HASMCs were treated with PM for designated times, and then the MMP-2 and MMP-9 protein expression and mRNA levels and cell migration were determined. (c) Cells were transfected with siRNA of scrambled (scrb), TLR2, TLR4, p47^phox^, p65, p50, or IL-6, followed by incubation with PM for 24 h. The MMP-2 and MMP-9 mRNA levels and cell migration were assessed. Data are presented as mean ± S.E.M. of three independent experiments. ^#^*P* < 0.01, as compared with control (b). ^#^*P* < 0.01, as compared with the cells exposed to PM plus scrb siRNA (c).

Considering the importance of VSMC migration in abnormal angiogenesis and vascular remodeling that are implicated in many vascular disorders [30], the migration of HASMCs in response to PM was investigated. MMPs are known as a family of zinc-dependent proteolytic enzymes that degrade various components of ECM, facilitating the progression of cell migration, among which MMP-2 and MMP-9 have been documented to associate with VSMC migration [30]. As shown in Figures 7(a) and 7(b), MMP-2 and MMP-9 protein expression and mRNA levels along with the migration index of PM-treated HASMCs were time-dependently enhanced as compared to control cells, suggesting the capacity of PM to induce MMP-2 and MMP-9 expression and subsequent cell migration in these vascular cells. To further determine the underlying mechanisms, transfection of HASMCs with siRNAs of TLR2, TLR4, p47^phox^, p65, p50, or IL-6 prior to PM stimulation was performed, then followed by MMP-2 and MMP-9 measurement as well as cell migration detection. Remarkably, all of the transfections were able to significantly reduce PM-induced MMP-2 mRNA levels, MMP-9 mRNA levels, and cell migration (Figure 7(c)). Our results indicated that PM could lead to MMP-2- and MMP-9-dependent HASMC migration which was regulated by TLR2, TLR4, NADPH oxidase, NF-*κ*B, and IL-6.

### 3.8. CORM-2 Decreases PM-Induced MMP-2 and MMP-9 Expression and Cell Migration in a TIMP-1/TIMP-2-Independent Manner

**Figure 8:**
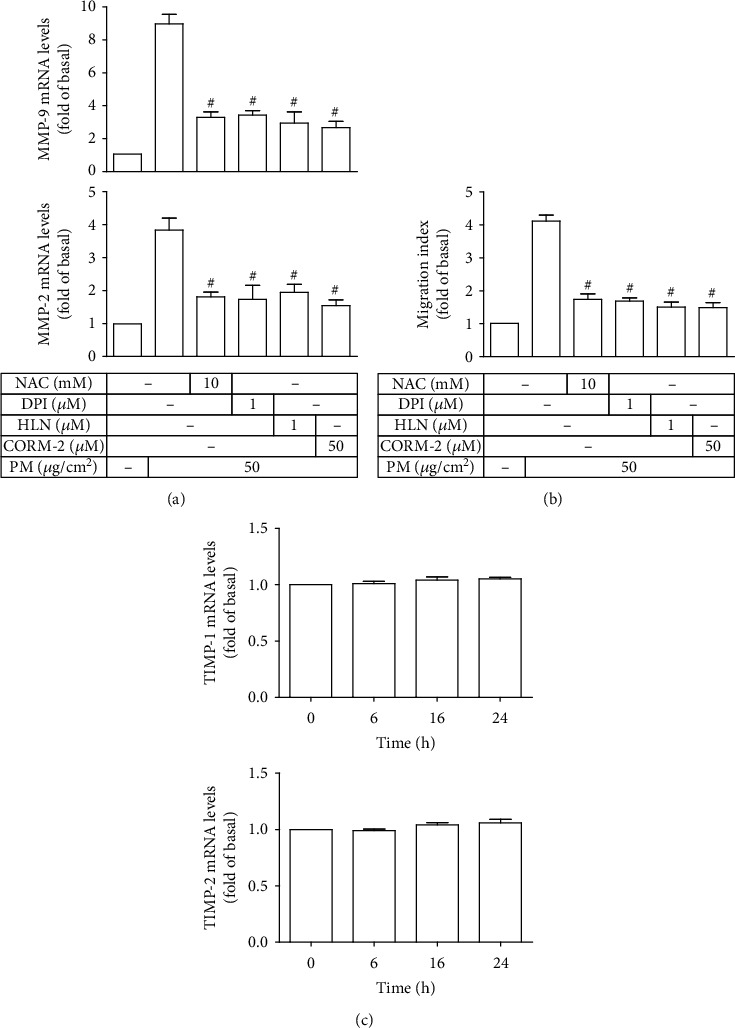
CORM-2 decreases PM-induced MMP-2 and MMP-9 expression and cell migration in HASMCs. (a, b) HASMCs were pretreated with NAC, DPI, HLN, or CORM-2, followed by incubation with PM for 24 h. The MMP-2 and MMP-9 mRNA levels and cell migration were determined. (c) Cells were treated with CORM-2 for designated times, and then the mRNA levels of TIMP-1 and TIMP-2 were measured. Data are presented as mean ± S.E.M. of three independent experiments. ^#^*P* < 0.01, as compared with the cells exposed to PM alone.

In line with the above results, Figures 8(a) and 8(b) show that pretreatment of HASMCs with inhibitor of ROS (NAC), NADPH oxidase (DPI), or NF-*κ*B (HLN) could markedly decrease PM-induced MMP-2 and MMP-9 mRNA levels as well as cell migration. Importantly, CORM-2 was observed to exert protective effects on HASMCs, as evidenced by the pronounced reduction in PM-induced MMP-2 mRNA levels, MMP-9 mRNA levels, and cell migration (Figures 8(a) and 8(b)). Given the fact that the activity of MMPs is tightly controlled by endogenous tissue inhibitors of MMPs (TIMPs) [29], the effects of CORM-2 on the expression of TIMP-1 and TIMP-2 were further examined. Surprisingly, TIMP-1 and TIMP-2 mRNA levels in HASMCs nearly remained following CORM-2 treatment over 24 h (Figure 8(c)). Thus, it seemed that CORM-2 could attenuate PM-induced HASMC migration in an MMP-2- and MMP-9-dependent manner but not TIMP-1 and TIMP-2.

### 3.9. CORM-2 Inhibits PM-Induced IL-6, MMP-2, MMP-9, VCAM-1, and ICAM-1 Expression in the Aorta Tissues of Mice

**Figure 9:**
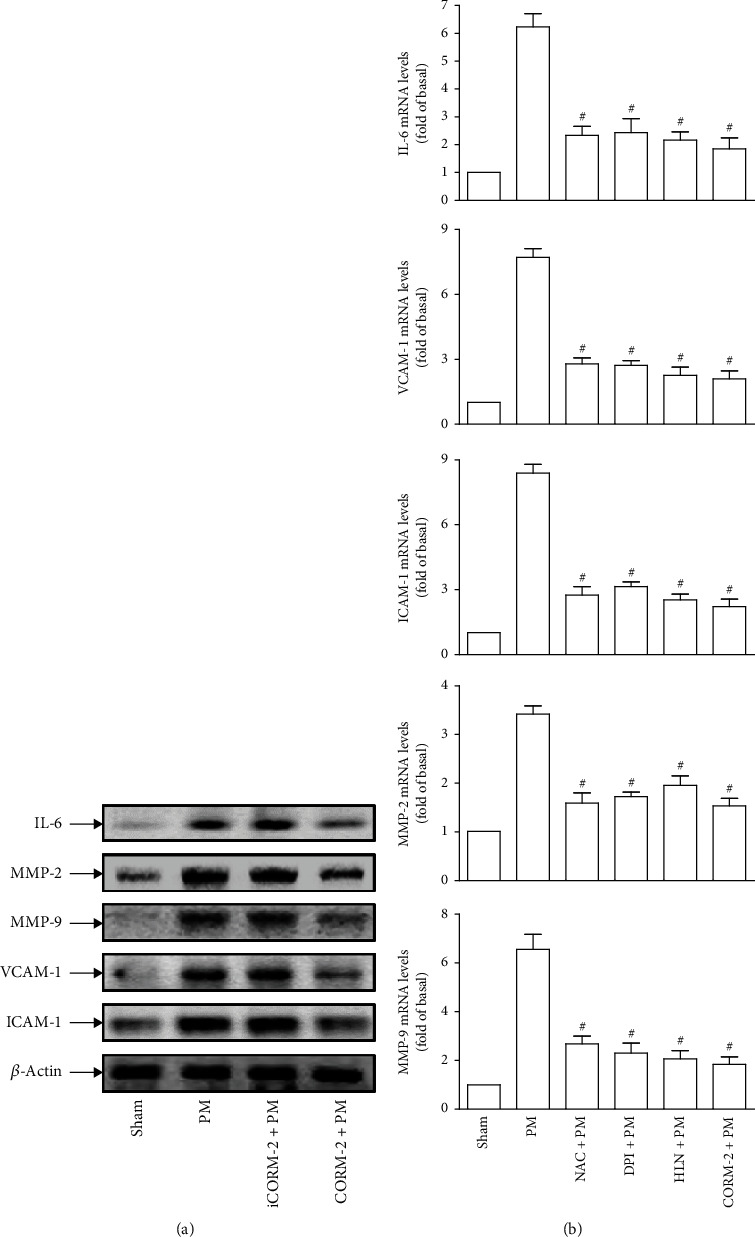
CORM-2 inhibits PM-induced IL-6, MMP-2, MMP-9, VCAM-1, and ICAM-1 expression in the aorta tissues of mice. (a) Mice were given i.v. one dose of iCORM-2 (8 mg/kg) or CORM-2 (8 mg/kg) prior to treatment with PM for 72 h. Preparation of thoracic aorta tissues was performed for subsequent analysis by Western blot to determine the protein expression of IL-6, MMP-2, MMP-9, VCAM-1, and ICAM-1. (b) Mice were given i.v. one dose of NAC, DPI, HLN, or CORM-2 prior to treatment with PM for 72 h. Preparation of thoracic aorta tissues was performed for subsequent analysis by real-time PCR to measure the mRNA levels of IL-6, MMP-2, MMP-9, VCAM-1, and ICAM-1. Data are presented as mean ± S.E.M. of three independent experiments. ^#^*P* < 0.01, as compared with the mice exposed to PM alone.

The passage of inhaled particles into the blood circulation, which may be associated with increased cardiovascular morbidity and mortality, was reported in humans [40]. Similarly, the translocation of intratracheally instilled PM from the lungs into the blood stream has been documented in several rodent experiments [41–43]. Therefore, in our animal experiments, PM was aspirated into the lungs of mice with or without pretreatment with iCORM-2 (8 mg/kg) or CORM-2 (8 mg/kg) administration, then sacrificed for the preparation of thoracic aorta tissues. Subsequently, the expression of molecules crucial for the development of vascular inflammation, including IL-6, MMP-2, MMP-9, VCAM-1, and ICAM-1, was measured. Western blot analysis showed that the protein expression of all examined molecules in PM-treated mice was evidently stronger than that in sham-operated mice (control), which was effectively inhibited by CORM-2 (Figure 9(a)). In contrast, iCORM-2 was unable to mitigate PM-induced protein expression of those molecules (Figure 9(a)), indicating the beneficial effects of exogenous CO released by CORM-2 rather than CORM-2 itself. The protective effects of CORM-2 was further confirmed by real-time PCR data in which PM-induced mRNA overexpression of IL-6, MMP-2, MMP-9, VCAM-1, and ICAM-1 was significantly decreased by CORM-2 administration (Figure 9(b)). Interestingly, mice given one dose of inhibitor of ROS (NAC), NADPH oxidase (DPI), or NF-*κ*B (HLN) prior to PM exposure also exhibited pronounced reduction in mRNA levels of all investigated molecules as compared to mice exposed to PM alone (Figure 9(b)). Altogether, *in vivo* data proposed that CORM-2-liberated CO may elicit protective effects on mice thoracic aorta tissues under PM insult by impeding IL-6, MMP-2, MMP-9, VCAM-1, and ICAM-1 expression through the inhibition of NADPH oxidase, ROS, and NF-*κ*B activities.

## 4. Discussion

**Figure 10:**
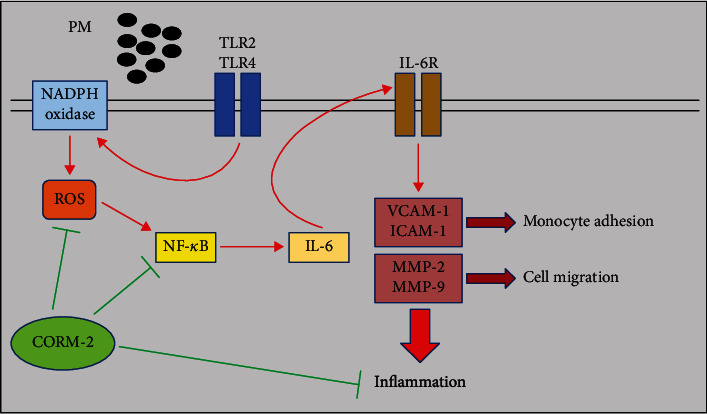
Proposed mechanistic scheme for anti-inflammatory effects of CO liberated from CORM-2 in PM-treated HASMC models. In HASMCs, PM can induce VCAM-1, ICAM-1, MMP-2, and MMP-9 expression through the TLR2 and TLR4/NADPH oxidase/ROS/NF-*κ*B/IL-6 pathway, thereby promoting monocyte adhesion and HASMC migration that are implicated in the development of inflammation. Importantly, CORM-2-liberated CO is able to prevent these inflammation-associated cellular events through the inhibition of the expression of relevant cell adhesion molecules and matrix metalloproteinases that is strictly regulated by a complex network of TLR2, TLR4, NADPH oxidase, ROS, NF-*κ*B, and IL-6 signaling molecules.

Inflammatory diseases of the aorta, with three broad categories consisting of atherosclerosis, atherosclerosis with excessive inflammation, and aortitis/periaortitis, are significant causes of morbidity and mortality [44]. The present study supports a novel model that exposure to PM can lead to an increase in the expression and secretion of IL-6 regulated by the TLR2 and TLR4/NADPH oxidase/ROS/NF-*κ*B signaling pathway, which then enhances the expression of cell adhesion molecules (ICAM-1 and VCAM-1) and MMPs (MMP-2 and MMP-9), thereby promoting monocyte adhesion and HASMC migration that are implicated in the development of aorta inflammation. Moreover, for the first time, our work provides evidence that exogenous CO released by CORM-2 can be a promising candidate for managing inflammatory diseases of the aorta induced by PM. In our experimental models, CORM-2 treatment effectively ameliorates aortic inflammatory responses under PM exposure, as evidenced by the reduction in the expression of proinflammatory cytokines, cell adhesion molecules, and gelatinases, accompanied with subsequent cellular events, as illustrated in Figure 10.

IL-6 is a pleiotropic cytokine that plays important roles in the pathogenesis of many CVDs [6]. A number of stimuli have been documented to associate with the IL-6 increase in vasculature, among which PM can be considered as one of cardiovascular risk factors. A recent meta-analysis consisting of 22 articles found a 4.66% increase in circulating IL-6 level per 10 *μ*g/m^3^ increment in PM2.5 concentration; in particular, the higher the PM2.5 level exposure, the more significant is the association with the IL-6 level [45]. Also, the pathological role of IL-6 in CVDs has been reported in various experimental models. For instance, the culture of human umbilical vein endothelial cells under PM2.5 exposure resulted in remarkable elevation in IL-6 secretion [46]. In mice, an instillation of 10 *μ*g PM less than 10 *μ*m in diameter (PM10) could enhance the prothrombotic tendency and thrombotic consequences which may be linked to the evident elevation of IL-6 levels, and IL-6 knockout mice were protected from PM-induced thrombotic events [47]. In line with literature, our data reported the pronounced increase in IL-6 expression and secretion in response to PM in cultured HASMCs. Moreover, PM was also associated with elevated IL-6 expression in thoracic aorta tissues dissected from exposed mice. These findings suggested the relationship between PM exposure and aorta inflammation via the IL-6 axis. It is noteworthy that the binding between IL-6 and the IL-6 receptor (IL-6R) is prerequisite for triggering a downstream cascade. The vasculature is responsive to IL-6 not only through classical IL-6 signaling via membrane-bound IL-6R but also through IL-6 transsignaling via soluble IL-6R, of which the latter seems to be the mainstream [9]. In this study, the IL-6R signaling was unidentified, demanding further investigation in the future.

It has been increasingly recognized that the majority of CVDs may result from the excessive accumulation of superoxide that is the precursor of most reactive species [9]. The phagocyte NADPH oxidase is acknowledged as a multicomponent enzyme responsible for superoxide production [10]. The identification of vascular oxidases other than those expressed in phagocytes has been an advance in the field of vascular biology. A number of pharmacologic and genetic approaches have demonstrated NADPH oxidase activity in vasculature [48]. The actions of NADPH oxidase subunits such as p47^phox^ and p67^phox^ which enhance oxidase activity and superoxide generation has been revealed in different vascular models as well [12, 49, 50]. In fact, our results also found that NADPH oxidase activity and its subunit p47^phox^ translocation were evidently yielded in cultured HASMCs in response to PM, significantly enhancing the intracellular ROS generation. It has been documented that the increase in vascular superoxide may modulate the expression and activation of proinflammatory molecules such as IL-6 in vasculature [9]. For instance, angiotensin II-induced IL-6 increase in VSMCs was reported to be inhibited in the presence of an NADPH oxidase inhibitor [11] or by the addition of the p47^phox^ antibody [12]. Consistently, in our study, pretreatment of HASMCs with pharmacologic inhibitors of ROS or NADPH oxidase effectively inhibited IL-6 secretion and expression in response to PM. Similar results were also obtained in cell knockdown of NADPH oxidase subunits such as p47^phox^ or p67^phox^. Thus, our findings suggested that NADPH oxidase-derived ROS may function as a signaling molecule occurring upstream of IL-6 expression induced by PM in aortic smooth muscle. Subsequently, NF-*κ*B was assumed in the current work as an important node linking vascular superoxide increase and IL-6 expression under PM exposure since it is an oxidative-sensitive transcription factor [51] and a major inducer of IL-6 [13]. Indeed, there were a transcriptionally pronounced upregulation of NF-*κ*B and a posttranslationally remarkable phosphorylation of the p65 subunit in PM-treated HASMCs, which was significantly inhibited by pretreatment with ROS or NADPH oxidase inhibitors. This implied that PM could induce NF-*κ*B activation in an NADPH oxidase-derived ROS-dependent manner. In addition, IL-6 secretion and expression in response to PM were evidently reduced in HASMC silencing of the p65 or p50 subunit, indicating the regulation of IL-6 by NF-*κ*B. Our previous study also detected the NADPH oxidase/ROS/NF-*κ*B/IL-6 signaling pathway in HASMCs but in response to angiotensin II [14]. The elevation of vascular smooth muscle IL-6 was reported to involve NF-*κ*B activities in other investigations as well [7, 52]. Therefore, our findings provided rational evidence linking NADPH oxidase-derived ROS and NF-*κ*B in the increase of IL-6 expression in aortic smooth muscle under PM insult. This was further confirmed by the *in vivo* data that mice receiving one dose of the pharmacologic inhibitor of either ROS, NADPH oxidase, or NF-*κ*B prior to PM exposure yielded a significant decrease of IL-6 mRNA levels in thoracic aorta tissues as compared to those exposed to PM alone.

Another line of evidences has supported the role of TLR signaling in the development of vascular inflammation [5]. Upon activation, TLRs further modulate the downstream NF-*κ*B signaling pathway, resulting in the production of proinflammatory cytokines and chemokines [15]. Increased expression of TLR2 and TLR4 was observed in various human inflammatory vasculopathies, including aortic lesions [53]. Experimentally, TLR2 stimulation was able to promote IL-6 expression and secretion in human adventitial fibroblasts isolated from the thoracic aorta dissected during heart transplantation, and the formation of vascular lesions following the *in vivo* application of the synthetic TLR2 ligand was not observed in TLR2 knockout mice [54]. In another study, exposure to microbial lipopolysaccharide, the prototypical TLR4 agonist, was identified as a potent inducer of IL-6 release in HASMCs as well as of NF-*κ*B activity in mouse aortic smooth muscle cells obtained from wild-type mice but not TLR4-deficient mice [55]. Herein, the data showed that PM exposure yielded evident TLR2 and TLR4 mRNA levels and protein expression in HASMCs. Moreover, our findings suggested the novel roles of both TLR2 and TLR4 in HASMCs, where IL-6 expression and secretion induced by PM were significantly inhibited in TLR2 or TLR4 knockdown cells, and so were intracellular ROS generation and NADPH oxidase activity. Altogether, IL-6 could be produced in aortic smooth muscle in response to PM through TLR2- and TLR4-dependent NADPH oxidase-derived ROS pathway triggering the activation of NF-*κ*B. Consistently, previous investigations indicated the involvement of both TLR2 and TLR4 in recognition of PM in Chinese hamster ovary cells, yielding remarkable IL-6 responses, and the application of a synthetic TLR4 antagonist was able to inhibit PM-induced IL-6 production from alveolar macrophages [56]. Another report also demonstrated the regulation of IL-6 expression in murine peritoneal macrophages exposed to PM through TLR2 and TLR4 [57].

In mammalian systems, heme oxygenase-1 (HO-1), known as a stress-inducible enzyme that degrades heme to CO and biliverdin, has emerged as an endogenous antioxidant and anti-inflammatory apparatus. It is acknowledged that CO plays important roles in anti-inflammatory activities behind HO-1 actions by modulating a wide range of transcription factor and inflammatory molecule pathways which underpin various inflammatory conditions [58]. A group of organo-metallo-compounds capable of delivering controlled amounts of exogenous CO to cells and tissues termed CORMs has been developed to mimic the function of HO-1 [58], among which CORM-2 has become of interest for its anti-inflammatory effects [22]. Importantly, the inhibitory effects of CORM-2 on inflammatory response may not be observed in the case of its inactive compound (iCORM-2) which does not release CO, as demonstrated in lipopolysaccharide-stimulated RAW264.7 murine macrophage models [32]. As anticipated, our results showed that the induction of IL-6 in HASMCs by PM was significantly reduced by the treatment with CORM-2 but not with iCORM-2, indicating that CO liberated from CORM-2 was the cause of IL-6 reduction. Indeed, the addition of the CO scavenger, hemoglobin, was reported to significantly reverse the cytoprotective effects of CORM-2 in angiotensin II-treated HASMCs [14]. In this study, *in vivo* data further showed that remarkable depletion of IL-6 mRNA levels and protein expression in thoracic aorta tissues were observed in mice pretreated with CORM-2 as compared to those exposed to PM alone, and such depletion was not detected in the iCORM-2 group. The exact mechanism for the reduction of PM-induced IL-6 by CORM-2 treatment remains ambiguous. Previous studies have revealed that the anti-inflammatory activities of CORM-2 may result from the ability of CO to modulate the NF-*κ*B transcription factor which regulates various proinflammatory molecules including IL-6. For instance, our earlier work reported that CORM-2 could attenuate angiotensin II-induced IL-6 expression in HASMCs by inhibiting NF-*κ*B activation [14]. In the current study, CORM-2 also downregulated NF-*κ*B activities, both transcriptionally and posttranslationally, in PM-treated HASMCs. However, CO treatment of hepatocytes was determined to activate NF-*κ*B *in vitro* and *in vivo* [59]. Thus, CO may exert dual effects on NF-*κ*B signaling, depending on the cell type. In addition to the NF-*κ*B transcription factor, oxidative stress due to ROS overproduction can be involved in an inflammatory cascade as well. Considering that CO has a high affinity for various heme-containing proteins in NADPH oxidase [58], anti-inflammatory activities of CORM-2 in the vasculature may further arise from the inhibition of vascular oxidase activity. Moreover, the chemical nature of transition metal carbonyls in CORM-2 may also modulate ROS production [58]. Indeed, NADPH oxidase was reported to be a target for CO derived from CORM-2, thus possibly modulating redox signaling in human airway smooth muscle cells [60]. Consistently, our data also showed that intracellular ROS generation and NADPH oxidase activity upon PM exposure were markedly reduced in CORM-2-pretreated HASMCs as compared to nonpretreated cells. Another possible signaling for the reduction of PM-induced IL-6 upon CORM-2 treatment in our study was the prevention of HASMCs from TLR2 and TLR4 expression, as evidenced by the apparent decrease of TLR2 and TLR4 mRNA levels in CORM-2-pretreated cells. In fact, the attenuation of inflammatory responses by CORM-2 through the inhibition of TLR2 and TLR4 was demonstrated in previous *in vitro* models such as human pulmonary alveolar epithelial cells or human aortic endothelial cells [23, 61]. Nevertheless, the underlying mechanisms for CORM-2-modulated TLR2 and TLR4 activities remain unclear. An *in vitro* study suggested the downregulation of the TLR4 signaling pathway in lipopolysaccharide-stimulated macrophages by HO-1-derived CO through the inhibition of TLR4 trafficking to membrane rafts in an NADPH oxidase-derived ROS-dependent manner, disrupting downstream signaling and cytokine production [62]. Collectively, the present study suggested a novel scheme where CORM-2 ameliorated PM-induced aorta inflammation by inhibiting IL-6 signaling that was strictly regulated through the interplay among TLR2 and TLR4, NADPH oxidase-derived ROS, and NF-*κ*B.

Investigation of downstream targets of the IL-6 signaling pathway may reveal more insights into the anti-inflammatory properties of CORM-2. It is intriguing that IL-6 has been reported to stimulate endothelial expression of adhesion molecules, including ICAM-1 and VCAM-1, thereby promoting leukocyte adherence and extravasation into the vasculature that are hallmarks of inflammation [9]. In addition to the endothelium, one investigation using the model of rat VSMCs showed that IL-6/interferon gamma could obviously incite VSMCs to secret ICAM-1 [63]. In our study, PM-induced IL-6 was indicated to trigger HASMCs to produce ICAM-1 and VCAM-1 since transfection of HASMCs with IL-6 siRNA could reverse the increase of those adhesion molecules in PM-exposed cells. Also, the upstream signaling molecules of IL-6, including NADPH oxidase, ROS, and NF-*κ*B, were demonstrated to regulate ICAM-1 and VCAM-1 expression in HASMCs upon PM exposure by using either pharmacologic inhibitor or siRNA transfection approaches. This was further supported by the *in vivo* finding that the reduction of PM-induced ICAM-1 and VCAM-1 expression was detected in thoracic aorta tissues dissected from mice given one dose of pharmacologic inhibitors of ROS, NADPH oxidase, or NF-*κ*B. Since IL-6 was produced in our HASMC models through a TLR2- and TLR4-dependent NADPH oxidase-derived ROS manner, it was rational to observe that the transfection of HASMCs with TLR2 or TLR4 siRNAs could decrease ICAM-1 and VCAM-1 expression in PM-treated cells. In addition, as important adhesion molecules for leukocyte adherence, pretreatment of HASMCs with an ICAM-1 or VCAM-1 neutralizing antibody was able to significantly inhibit the adherence of human monocytic cells THP-1 following PM exposure. Besides, the regulatory roles of NADPH oxidase, ROS, and NF-*κ*B on monocyte adherence were identified by using a pharmacologic inhibitor approach as well. Collectively, our results provided novel insight into the downstream mechanism of the IL-6 signaling pathway in PM-induced aorta inflammation, that is, ICAM-1 and VCAM-1-dependent monocyte adhesion. Interestingly, the benefits of CORM-2 in mitigating PM-induced ICAM-1 and VCAM-1 expression through the inhibition of the IL-6 signaling pathway were demonstrated in both HASMCs and mice models, thereby inhibiting monocyte adherence *in vitro*. Consistently, the protective effects of CO released from CORM-2 through targeting ICAM-1 and/or VCAM-1 have been documented in previous studies using different experimental models [26–28].

Earlier evidences have indicated that IL-6 can promote the migration of VSMCs, which is another hallmark of progression of vascular inflammation [9]. The roles of MMP-2 and MMP-9 in VSMC migration have also been widely documented [30]. As a result, MMP-2- and MMP-9-dependent vascular cell migration can be another downstream mechanism of the IL-6 signaling pathway in parallel to monocyte adherence, thereby serving as an additional target of CORM-2. The first evidence for the effect of CO on MMP expression and activity was reported in 2005. It was found that CORM-2, but not iCORM-2 which did not contain CO groups, could inhibit both MMP-1 and MMP-2 activities in the human lung epithelial cell line A549 [64]. In this study, PM was found to induce both MMP-2 and MMP-9 expression in HASMCs along with subsequent HASMC migration. Similarly, our previous studies also reported that CORM-2 could attenuate angiotensin II-induced HASMC migration [14] or *Staphylococcus aureus*-induced human aortic endothelial cell migration [50] by inhibiting MMP-9 in an IL-6-dependent manner. Next, by using pharmacologic inhibitor-combined siRNA transfection approaches, TLR2, TLR4, NADPH oxidase, NF-*κ*B, and IL-6 were demonstrated to regulate MMP-2- and MMP-9-dependent HASMC migration in response to PM. These findings were further confirmed by the results that thoracic aorta tissues obtained from PM-exposed mice also exhibited significantly stronger expression of MMP-2 and MMP-9 which were alleviated by pretreatment with inhibitors of ROS, NADPH oxidase, or NF-*κ*B. Importantly, our study suggested a novel scheme where CORM-2 could inhibit both MMP-2 and MMP-9 expression through the disruption of the IL-6 signaling pathway *in vitro* and *in vivo*, thereby reducing HASMC migration upon PM stimulation. Furthermore, the effects of CORM-2 on TIMPs which are regulators of MMPs were evaluated. Our finding that CORM-2 was unable to regulate TIMP-1 and TIMP-2 was somewhat surprising since a correlation between IL-6 levels with TIMP-1 and TIMP-2 expression was reported previously [65]. This suggested that CORM-2 can regulate MMP-2 and MMP-9 expression and vascular cell migration in a TIMP-1- and TIMP-2-independent manner.

## 5. Conclusions

Our work is an effort to explore the molecular and cellular mechanisms that determine the beneficial effects of CORM-2. To sum up, exogenous CO liberated from CORM-2 has protective activities against PM-induced aorta inflammation through the inhibition of the TLR2 and TLR4/NADPH oxidase/ROS/IL-6 signaling pathway, thereby reducing the expression of ICAM-1 and VCAM-1 as well as MMP-2 and MMP-9 that was followed by monocyte adherence and aortic smooth muscle cell migration. Together with *in vivo* data, our study clearly highlights the potential of CORM-2 as a promising candidate for the impediment of inflammatory diseases of the aorta. Further investigations into the therapeutic applicability of CORM-2 are highly demanded.

## Data Availability

The data used to support the findings of this study are included within the article.

## Abbreviations

APO: Apocynin
CO: Carbon monoxide
CORMs: Carbon monoxide-releasing molecules
CVDs: Cardiovascular diseases
DPI: Diphenyleneiodonium chloride
ECM: Extracellular matrix
HASMCs: Human aortic smooth muscle cells
HLN: Helenalin
HO-1: Heme oxygenase-1
ICAM-1: Intercellular adhesion molecule-1
iCORM-2: Inactive CORM-2
IL-6: Interleukin-6
IL-6R: IL-6 receptor
MMPs: Matrix metalloproteinases
NAC: N-Acetyl-L-cysteine
NADPH: Nicotinamide adenine dinucleotide phosphate
NF-*κ*B: Nuclear factor-kappa B
PM: Particulate matter
ROS: Reactive oxygen species
siRNA: Small interfering RNA
TIMPs: Tissue inhibitors of MMPs
TLRs: Toll-like receptors
VCAM-1: Vascular cell adhesion molecule-1
VSMCs: Vascular smooth muscle cells.
